# Incidence and Prognostic Significance of Arrhythmia in Acute Myocardial Infarction Presentation: An Observational Study

**DOI:** 10.7759/cureus.71564

**Published:** 2024-10-15

**Authors:** Pradeep Kurmi, Ankit Patidar, Sudarshan Patidar, Utsav Yadav

**Affiliations:** 1 Cardiology, Super Speciality Hospital, Mahatma Gandhi Memorial Medical College (MGMMC), Indore, IND; 2 Medicine, Super Speciality Hospital, Super Speciality Hospital, Mahatma Gandhi Memorial Medical College (MGMMC), Indore, IND; 3 Medicine, Peoples Medical College, Bhopal, IND

**Keywords:** cardiac arrhythmia, cardio vascular disease, mortality, myocardial infarction, risk factors

## Abstract

Background

Arrhythmias are well-recognized complications of acute myocardial infarction (AMI) and are an important risk factor for mortality in both men and women across a wide age range.

Aim

This study aims to analyze the incidence of arrhythmia in patients with AMI with respect to age, gender distribution, and location of AMI and also to evaluate the prognostic factors of mortality in patients with AMI.

Methods

This prospective, observational, and cross-sectional study included 300 patients admitted within an hour of the presentation of AMI at a Super Speciality Hospital, MGMMC (Mahatma Gandhi Memorial Medical College), Indore, after fulfilling the inclusion criteria. Clinical features, elevated cardiac biomarkers, and an electrocardiogram guided the diagnosis of AMI and arrhythmia.

Results

Of the total of 300 patients, the majority were male, 280 (93.4%), with a mean age of 57.48 ± 13.48 years. Prevalent risk factors included obesity, 195 (65%); diabetes mellitus, 185 (61.66%); hypertension, 181 (60.33%); smoking, 114 (38%); alcohol consumption, 123 (41%); and hypercholesterolemia, 207 (69%). Among 152 patients with arrhythmia, obesity, ischemic heart disease, diabetes, hypertension, smoking, and alcohol were more prevalent compared to those without arrhythmia. The arrhythmia incidence was higher in 143 (51.07%) male patients. Out of 37 mortality cases, 29 were associated with arrhythmia. Mortality was highest in extensive anterior wall acute myocardial infarction (EAWMI), 14 (37.24%), and inferior wall myocardial infarction (IWMI), 13 (35.14%).

Conclusion

In conclusion, arrhythmia was prevalent in the age group of 45-54 years and among patients with EAWMI. Mortality was significantly associated with arrhythmia and was highest in elderly patients with EAWMI and IWMI. These findings underscore the importance of risk stratification and targeted management strategies.

## Introduction

Acute myocardial infarction (AMI) is characterized by myocardial cell necrosis, which can lead to a variety of complications, including arrhythmic, mechanical, and inflammatory sequelae. Approximately 90% of patients with AMI develop some form of cardiac arrhythmia. In 25% of patients, these rhythm abnormalities manifest within the first 24 hours, and the risk of ventricular fibrillation is highest in the first hour [1,2].

According to data reported by the American Heart Association in 2015, the 30-day mortality rates for patients with myocardial infarction range from 7.8% to 11.4% [3]. Cardiac arrhythmia frequently complicates the clinical management of AMI. Multiple studies have established a significant association between different arrhythmias and higher morbidity and mortality in patients with AMI [4-7]. Despite the rapid advances in revascularization therapies over the last decades, managing complex arrhythmia remains challenging, particularly during the acute phase of myocardial infarction (MI) [8]. The primary mechanisms of arrhythmia during the acute phase of coronary occlusion involve re-entry, which occurs due to the uneven electrical properties of the ischemic myocardium. Additionally, the cellular electrophysiological process for reperfusion-related arrhythmia involves the removal of accumulated ions like lactate, potassium, and harmful metabolic substances from the ischemic area [9]. Also, arrhythmia can occur as a direct consequence of the ischemic injury to the myocardium, electrolyte imbalances, or as a result of the reperfusion therapy itself, such as primary percutaneous coronary intervention (PCI) or thrombolytic therapy [10].

Arrhythmia during the golden hours of MI, defined as an hour after MI, can have severe consequences, including hemodynamic instability, impaired cardiac output, and an increased risk of sudden cardiac death. Certain types of arrhythmias, such as ventricular fibrillation, are particularly life-threatening, with mortality rates as high as 80% if left untreated [11]. Early recognition and appropriate management of these arrhythmias are crucial in preventing lethal outcomes and improving patient survival.

Despite the significance of this issue, there is a paucity of comprehensive studies examining the clinical profile of arrhythmia in AMI patients during the golden hours. This study aims to bridge this gap by providing a comprehensive analysis of the clinical profile of arrhythmia in patients with AMI during the golden hours by identifying the prevalence and associated risk factors of arrhythmia, as well as evaluating the mortality rates and its prognostic factors.

## Materials and methods

This was a prospective, observational, cross-sectional, and single-center study conducted at the Cardiology Department of Super Speciality Hospital, MGMMC, Indore, in India, from May 1, 2023, to June 30, 2024. The study included 300 patients of both genders, aged 35-85 years, who had clinical features of AMI and had arrhythmia both pre- and post-thrombolysis. The diagnosis of AMI was based on the presenting symptoms, electrocardiography (ECG or EKG) reflecting ST segment deviation, and elevated cardiac biomarkers, i.e., CK-MB and troponin-T. The study excluded patients with preexisting MI, other cardiac diseases, or a prior history of any cardiac intervention. The study was conducted in accordance with the Declaration of Helsinki and after receiving approval from the institutional ethics committee. Informed consent was obtained from all the participants.

Myocardial infarction often happens due to insufficient oxygen reaching the heart muscle, typically caused by reduced blood flow in one or more coronary arteries. Such blockages can result from various issues with arterial plaques, including ruptures, erosion, cracks, or coronary artery dissection [12]. Cardiac arrhythmias encompass various conditions where the heart's electrical signaling is disrupted, causing it to beat irregularly - either too fast or too slow compared to its normal rhythm [13].

Demographic and clinical characteristics of the patients, such as age (years), weight (kg), height, gender, obesity, smoking, diabetic mellitus, family history of coronary artery disease, and hypertension, were recorded. Laboratory investigations were performed for all the enrolled patients, including complete blood count, renal function tests, troponin T, fasting and postprandial blood sugar, liver function tests, and lipid profiles. Cardiac arrhythmia was diagnosed on the basis of ECG changes. All the patients were monitored, and the incidence of cardiac arrhythmia and outcomes were recorded.

Data were analyzed using SPSS software version 23.0 (IBM Corp., Armonk, NY). Numeric variables were presented as mean ± standard deviation. Frequency and percentages were calculated for categorical variables. Risk factors, angiographic and procedural characteristics, and outcome (in-hospital mortality) were compared with the incidence of arrhythmia using the chi-square test or Fisher’s exact test. A p-value of <0.05 was taken as the criterion of statistical significance.

## Results

In this study, a total of 300 patients were included, with a mean age of 57.48 ± 13.48 years and predominantly male, 280 (93.4%). The study participants exhibited risk factors, where a significant proportion of the patients had obesity, 195 (65%); diabetes mellitus, 185 (61.66%); hypertension, 181 (60.33%); smoking history, 114 (38%); alcohol consumption, 123 (41%); and hypercholesterolemia, 207 (69%). Only a small number, 9 (3%), of the participants were menopausal women. The location of MI varied among the patients. Anterior lateral MI (ALMI) was observed in 18.33% (55) of the cases, anterior wall MI (AWMI) in 29.33% (88), extensive anterior wall MI (EAWMI) in 19.34% (58), and inferior wall MI (IWMI) in 33% (99) of the participants (Table 1).

**Table 1:** Baseline characteristics of the study population Data are represented as mean ± standard deviation and n (%) as appropriate. ALMI: anterior lateral MI; AWMI: anterior wall MI; DM: diabetes mellitus; EAWMI: extensive anterior wall MI; IWMI: inferior wall MI; MI: myocardial infarction.

Characteristics	N= 300 patients
Age (years, mean ± SD)	57.48 ± 13.48
Male, n (%)	280 (93.4%)
Risk factors	
Obesity, n (%)	195 (65%)
DM, n (%)	185 (61.66%)
Hypertension, n (%)	181 (60.33%)
Smoking, n (%)	114 (38%)
Alcohol, n (%)	123 (41%)
Menopause, n (%)	9 (3%)
Hypercholesterolemia, n (%)	207 (69%)
Location of MI	
ALMI, n (%)	55 (18.33%)
AWMI, n (%)	88 (29.33%)
EAWMI, n (%)	58 (19.34%)
IWMI, n (%)	99 (33%)

Comparison of risk factors between those who developed arrhythmia and those who did not, as described in Table 2, revealed a higher prevalence of obesity (67.8% vs. 62.2%, p=0.02), diabetes mellitus (68.4% vs. 54.7%, p=0.001), hypertension (67.1% vs. 53.4%, p=0.04), smoking (36.8% vs. 39.2%, p=0.012), and alcohol consumption (43.4% vs. 38.5%, p=0.023) in the arrhythmia group compared to the non-arrhythmia group. Menopausal status did not differ significantly between groups. Hypercholesterolemia was more prevalent in the non-arrhythmia group (70.3% vs. 67.8%, p=0.014).

**Table 2:** Data are represented as n (%) as appropriate. P-value ≤ 0.05 is considered significant. DM: diabetes mellitus.

Risk factors	Arrhythmia (N=152 patients)	No arrhythmia (N=148 patients)	P-value
Obesity, n (%)	103	92	0.02
DM, n (%)	104	81	0.001
Hypertension, n (%)	102	79	0.04
Smoking, n (%)	56	58	0.012
Alcohol, n (%)	66	57	0.023
Menopausal, n (%)	3	6	0.21
Hypercholesterolemia, n (%)	103	104	0.014

Among the 37 mortality cases, older age groups were more affected, with 16 patients (43.24%) aged between 65 and 74 years and 13 patients (35.14%) aged between 75 and 84 years. Patients among those who experienced mortality, 26 patients (70.27%) were male, and 11 patients (29.73%) were female (Table 3).

**Table 3:** Demographic and clinical characteristics of patients with mortality Data are represented as n (%) as appropriate. ALMI: anterior lateral MI; AWMI: anterior wall MI; EAWMI: extensive anterior wall MI; IWMI: inferior wall MI; MI: myocardial infarction.

Variables	Mortality (N=37 patients)
Age	
35–44 years, n (%)	1 (2.70%)
45–54 years, n (%)	4 (10.81%)
55–64 years, n (%)	3 (8.11%)
65–74 years, n (%)	16 (43.24%)
75–84 years, n (%)	13 (35.14%)
Gender	
Male, n (%)	26 (70.27%)
Female, n (%)	11 (29.73%)
Location of MI	
ALMI, n (%)	2 (5.4%)
AWMI, n (%)	8 (21.62%)
EAWMI, n (%)	14 (37.84%)
IWMI, n (%)	13 (35.14%)

**Figure 1:**
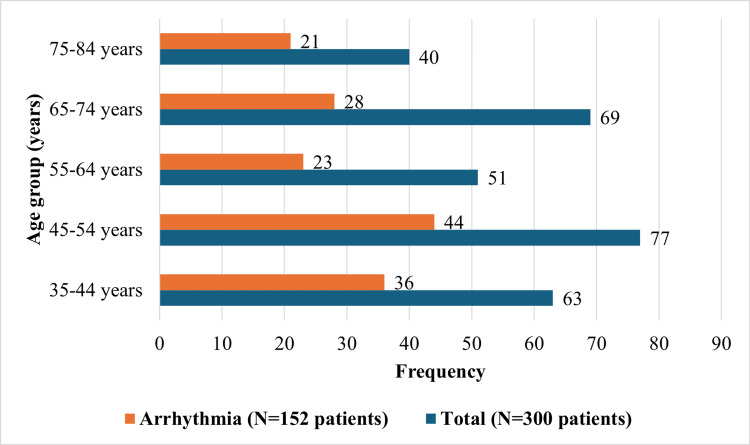
Association between age and the occurrence of arrhythmia in patients with myocardial infarction

Patients with EAWMI had the highest mortality rate (37.84%), followed by IWMI (35.14%). The association between age and the occurrence of arrhythmia in patients with myocardial infarction is depicted in Figure 1.

**Figure 2:**
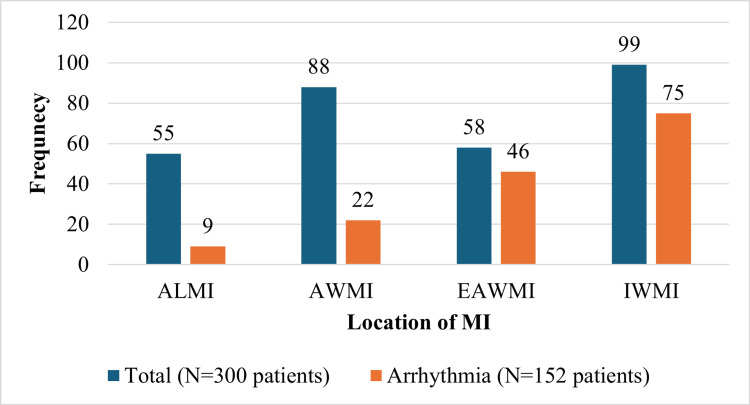
Association between location of myocardial infarction and the occurrence of arrhythmia in patients with myocardial infarction ALMI: anterior lateral MI; AWMI: anterior wall MI; EAWMI: extensive anterior wall MI; IWMI: inferior wall MI.

Among the male patients, n=143 (51.07%) developed arrhythmia, while among the female patients, n=9 (45%) experienced arrhythmia. The incidence of arrhythmia was higher in the age group between 45 and 54 years, with 44 patients out of 77 (57.14%). Figure 2 illustrates the association between the location of MI and the occurrence of arrhythmia in the study patients. Patients with EAWMI have a high incidence of arrhythmia, followed by IWMI.

## Discussion

The present study highlights the substantial burden of arrhythmia in patients with AMI, affecting over half (51%) of the study population. This is consistent with previous reports indicating that arrhythmia is a common complication in AMI, occurring in up to 90% of patients [14,15]. In a study by Mehta et al., a total of 89.1% of the patients with AMI were observed to have arrhythmia [14]. However, the variance in the incidence rate is likely due to differences in the study populations and individual criteria for diagnosing arrhythmia.

Aging is associated with increased fibrosis and conduction abnormalities in the myocardium, predisposing to reentrant arrhythmia [14]. The higher incidence of arrhythmia in older age groups is consistent with previous studies. In prior research, the incidence of atrial fibrillation after AMI ranged from 6-21% and increased with age [16]. The GUSTO-III trial reported the incidence of ventricular tachycardia/fibrillation was 7.5% in patients <65 years old compared to 12.5% in those ≥75 years [15].

However, some studies suggest that females may be an independent risk factor for post-MI arrhythmia. One study found that women had a higher risk of atrial fibrillation (HR 1.16) and ventricular tachycardia/fibrillation (HR 1.24) after AMI. Potential mechanisms might include differences in autonomic tone, inflammation, and hormonal factors between both genders [17].

The findings of this study align with prior research, highlighting the importance of infarct location and size in the development of arrhythmia after AMI. A study by Lopes et al. [18] reported that patients with AWMI had a significantly higher risk of developing ventricular arrhythmia compared to those with IWMI (OR=2.1, 95% CI: 1.4-3.1, p<0.0001). Another study by Gheeraert et al. found that the incidence of ventricular tachycardia was significantly higher in patients with AWMI (32.4%) compared to those with IWMI (17.6%, P<0.001) [19]. However, in our study, patients with EAWMI exhibited the highest incidence of arrhythmia, followed by IWMI.

The association between arrhythmia and mortality in AMI patients can be attributed to several potential mechanisms. Arrhythmia can lead to hemodynamic instability, impaired cardiac output, and an increased risk of sudden cardiac death, particularly in the case of life-threatening arrhythmia such as ventricular fibrillation. A study by Hassan et al. reported a significantly higher in-hospital mortality rate among AMI patients with ventricular arrhythmia compared to those without arrhythmia (12.9% vs. 3.8%, p<0.001) [20]. The highest mortality rates in our study were observed in patients with EAWMI (37.84%) and IWMI (35.14%), reinforcing the importance of early recognition and appropriate management of arrhythmia in these high-risk subgroups.

Limitations

This study has several limitations that should be acknowledged. First, it was a single-center study conducted at a tertiary care hospital in India, including a small sample size; hence, the generalizability of the findings to other geographic regions or healthcare settings is limited. Second, the study relied on ECG (electrocardiogram) findings and cardiac biomarkers to diagnose arrhythmia and MI, which may have limited sensitivity and specificity compared to more advanced diagnostic modalities. Also, data on the specific types and severities of arrhythmia were not collected, which could have provided additional insights into their prognostic implications. Finally, the study did not account for potential confounding factors, such as comorbidities, medications, or the timing and type of reperfusion therapy, which could have influenced the incidence and outcomes of arrhythmia.

## Conclusions

In conclusion, we found a high incidence of arrhythmia, particularly between the age group of 45-54 years, males, and those with EAWMI. The presence of arrhythmia was significantly associated with traditional cardiovascular risk factors, such as obesity, diabetes, hypertension, smoking, and alcohol consumption. Importantly, arrhythmia was strongly associated with in-hospital mortality, with the highest mortality rates observed in elderly patients with EAWMI and IWMI. Hence, close monitoring and prompt treatment of arrhythmia, especially in high-risk subgroups, may improve clinical outcomes and reduce mortality associated with AMI.

## References

[1] Thygesen K, Alpert JS, White HD (2007). Universal definition of myocardial infarction. Circulation.

[2] Marangmei L, Singh SK, Devi KB, Raut SS, Chongtham DS, Singh KB (2014). Profile of cardiac arrhythmia in acute myocardial infarction patients within 48 hours of admission: a hospital based study at RIMS Imphal. J Med Soc.

[3] Mozaffarian D, Benjamin EJ, Go AS (2015). Heart disease and stroke statistics--2015 update: a report from the American Heart Association. Circulation.

[4] Jabre P, Roger VL, Murad MH, Chamberlain AM, Prokop L, Adnet F, Jouven X (2011). Mortality associated with atrial fibrillation in patients with myocardial infarction: a systematic review and meta-analysis. Circulation.

[5] Henkel DM, Witt BJ, Gersh BJ, Jacobsen SJ, Weston SA, Meverden RA, Roger VL (2006). Ventricular arrhythmias after acute myocardial infarction: a 20-year community study. Am Heart J.

[6] Shacham Y, Leshem-Rubinow E, Steinvil A, Keren G, Roth A, Arbel Y (2015). High degree atrioventricular block complicating acute myocardial infarction treated with primary percutaneous coronary intervention: incidence, predictors and outcomes. Isr Med Assoc J.

[7] Obayashi Y, Shiomi H, Morimoto T (2021). Newly diagnosed atrial fibrillation in acute myocardial infarction. J Am Heart Assoc.

[8] Frampton J, Ortengren AR, Zeitler EP (2023). Arrhythmias after acute myocardial infarction. Yale J Biol Med.

[9] Antman EM (2005). ST-elevation myocardial infarction: management. Braunwald's Heart Disease.

[10] Scirica BM (2010). Acute coronary syndrome: emerging tools for diagnosis and risk assessment. J Am Coll Cardiol.

[11] Myerburg RJ, Castellanos A (2006). Emerging paradigms of the epidemiology and demographics of sudden cardiac arrest. Heart Rhythm.

[12] Anderson HV, Masri SC, Abdallah MS (2022). ACC/AHA key data elements and definitions for chest pain and acute myocardial infarction: a report of the American Heart Association/American. Circ Cardiovasc Qual Outcomes.

[13] Hassan SU, Mohd Zahid MS, Abdullah TA, Husain K (2022). Classification of cardiac arrhythmia using a convolutional neural network and bi-directional long short-term memory. Digit Health.

[14] Mehta RH, Starr AZ, Lopes RD (2009). Incidence of and outcomes associated with ventricular tachycardia or fibrillation in patients undergoing primary percutaneous coronary intervention. JAMA.

[15] Al-Khatib SM, Stebbins AL, Califf RM (2003). Sustained ventricular arrhythmias and mortality among patients with acute myocardial infarction: results from the GUSTO-III trial. Am Heart J.

[16] Patel MR, Chen AY, Peterson ED (2006). Prevalence, predictors, and outcomes of patients with non-ST-segment elevation myocardial infarction and insignificant coronary artery disease: results from the Can Rapid risk stratification of Unstable angina patients Suppress ADverse outcomes with Early implementation of the ACC/AHA Guidelines (CRUSADE) initiative. Am Heart J.

[17] Kragholm KH, Lindgren FL, Zaremba T (2021). Mortality and ventricular arrhythmia after acute myocarditis: a nationwide registry-based follow-up study. Open Heart.

[18] Lopes RD, Elliott LE, White HD (2009). Antithrombotic therapy and outcomes of patients with atrial fibrillation following primary percutaneous coronary intervention: results from the APEX-AMI trial. Eur Heart J.

[19] Gheeraert PJ, Henriques JP, De Buyzere ML (2000). Out-of-hospital ventricular fibrillation in patients with acute myocardial infarction: coronary angiographic determinants. J Am Coll Cardiol.

[20] Newby KH, Thompson T, Stebbins A, Topol EJ, Califf RM, Natale A (1998). Sustained ventricular arrhythmias in patients receiving thrombolytic therapy: incidence and outcomes. Circulation.

